# Risk factors for developing severe COVID-19 in China: an analysis of disease surveillance data

**DOI:** 10.1186/s40249-021-00820-9

**Published:** 2021-04-12

**Authors:** Meng-Jie Geng, Li-Ping Wang, Xiang Ren, Jian-Xing Yu, Zhao-Rui Chang, Can-Jun Zheng, Zhi-Jie An, Yu Li, Xiao-Kun Yang, Hong-Ting Zhao, Zhong-Jie Li, Guang-Xue He, Zi-Jian Feng

**Affiliations:** 1 Division of Infectious Diseases, Chinese Center for Disease Control and Prevention, Beijing, China; 2 National Institute for Communicable Disease Control and Prevention, Chinese Center for Disease Control and Prevention, Beijing, China; 3 National Immunization Program, Chinese Center for Disease Control and Prevention, Beijing, China; 4 National Institute for Viral Disease Control and Prevention, Chinese Center for Disease Control and Prevention, Beijing, China

**Keywords:** COVID-19, Severe case, Non-severe case, Risk factor

## Abstract

**Background:**

COVID-19 has posed an enormous threat to public health around the world. Some severe and critical cases have bad prognoses and high case fatality rates, unraveling risk factors for severe COVID-19 are of significance for predicting and preventing illness progression, and reducing case fatality rates. Our study focused on analyzing characteristics of COVID-19 cases and exploring risk factors for developing severe COVID-19.

**Methods:**

The data for this study was disease surveillance data on symptomatic cases of COVID-19 reported from 30 provinces in China between January 19 and March 9, 2020, which included demographics, dates of symptom onset, clinical manifestations at the time of diagnosis, laboratory findings, radiographic findings, underlying disease history, and exposure history. We grouped mild and moderate cases together as non-severe cases and categorized severe and critical cases together as severe cases. We compared characteristics of severe cases and non-severe cases of COVID-19 and explored risk factors for severity.

**Results:**

The total number of cases were 12 647 with age from less than 1 year old to 99 years old. The severe cases were 1662 (13.1%), the median age of severe cases was 57 years [Inter-quartile range(IQR): 46–68] and the median age of non-severe cases was 43 years (IQR: 32–54). The risk factors for severe COVID-19 were being male [adjusted odds ratio (a*OR*) = 1.3, 95% *CI:* 1.2–1.5]; fever (a*OR* = 2.3, 95% *CI:* 2.0–2.7), cough (a*OR* = 1.4, 95% *CI:* 1.2–1.6), fatigue (a*OR* = 1.3, 95% *CI:* 1.2–1.5), and chronic kidney disease (a*OR* = 2.5, 95% *CI:* 1.4–4.6), hypertension (a*OR* = 1.5, 95% *CI:* 1.2–1.8) and diabetes (a*OR* = 1.96, 95% *CI:* 1.6–2.4). With the increase of age, risk for the severity was gradually higher [20–39 years (a*OR* = 3.9, 95% *CI:* 1.8–8.4), 40–59 years (a*OR* = 7.6, 95% *CI:* 3.6–16.3), ≥ 60 years (a*OR* = 20.4, 95% *CI:* 9.5–43.7)], and longer time from symtem onset to diagnosis [3–5 days (a*OR* = 1.4, 95% *CI:* 1.2–1.7), 6–8 days (a*OR* = 1.8, 95% *CI:* 1.5–2.1), ≥ 9 days(a*OR* = 1.9, 95% *CI:* 1.6–2.3)].

**Conclusions:**

Our study showed the risk factors for developing severe COVID-19 with large sample size, which included being male, older age, fever, cough, fatigue, delayed diagnosis, hypertension, diabetes, chronic kidney diasease. Based on these factors, the severity of COVID-19 cases can be predicted. So cases with these risk factors should be paid more attention to prevent severity.

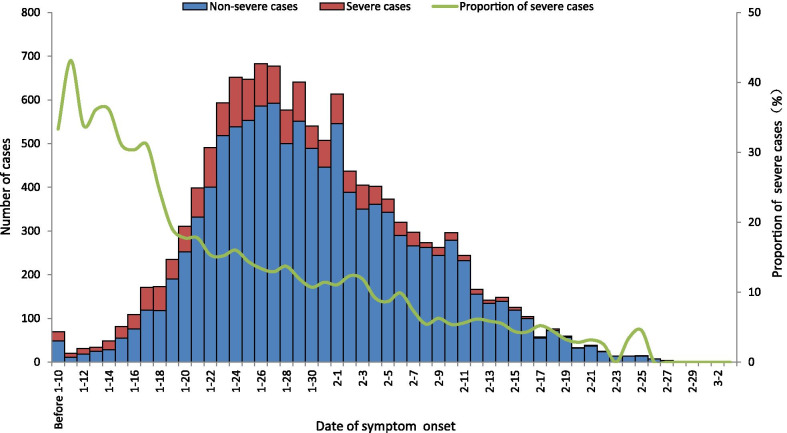

## Background

Coronavirus disease 2019 (COVID-19) is caused by severe acute respiratory syndrome coronavirus 2 (SARS-CoV-2) and is transmitted by respiratory droplets and contacts. Sources of infection are cases and asymptomatic infections, infectivity starts from the incubation period and becomes relatively strong within five days after symptom onset, the populations are generally susceptible to infection [1]. The contagiousness of SARS-CoV-2 is higher than severe acute respiratory syndrome coronavirus (SARS-CoV) and Middle East respiratory syndrome coronavirus (MERS-CoV), and the scale of morbidity and mortality of COVID-19 are far greater than Severe Acute Respiratory Syndrome (SARS) and Middle East Respiratory Syndrome (MERS) [2–4]. The World Health Organization (WHO) declared COVID-19 a public health emergency of international concern on January 30, 2020 [5]. COVID-19 has brought great burden on the public health worldwide; as of December 27, 2020, more than 210 countries and regions worldwide have reported 79.2 million cases and more than 1.7 million deaths [6]. The National Health Commission of the People’s Republic of China made COVID-19 a Class B notifiable infectious diseases, but managed COVID-19 as a Class A infectious disease [7, 8].

COVID-19 causes lots of symptoms, including fever, cough, fatigue, and the clinical spectrum of COVID-19 pneumonia ranges from mild to critical. More than 80% infected individuals are asymptomatic or develop mild upper respiratory tract symptoms were reported [9], and parts of cases have severe courses that can include dyspnea, hypoxemia, Acute Respiratory Distress Syndrome (ARDS), shock, and even death. Mild and moderate cases can recover after symptomatic treatment, but severe and critical patients require early intervention, respiratory support and sometimes intensive care unit (ICU) care [10]. The case fatality rates of severe and critical cases are relatively high, critical patients witness significantly higher mortality which is up to 49.0%, by contrast with 2.3% for overall COVID-19 patients [9, 11]. Due to the higher mortality of severe cases, there is a need to explore the risk factors for severity, prevent developing severe cases, followed by reducing the case fatality rate.

Most studies on exploring risk factors for COVID-19 severity in China were with limited sample size and most focused on influence of clinical characteristics and treatments on severity [12–14]. Whereas comparisons of severe and non-severe cases and the influences of early symptoms on predicating progression of COVID-19 need to be further explored.

 Wuhan reached the highest peak of the COVID-19 outbreak around February 12, 2020, diagnosis and treatment may have been delayed because the medical resources for COVID-19 were limited at that time [15]. During early stage, the COVID-19 case fatality rate in Hubei Province was higher than other provinces in mainland China [16, 17]. Severity of COVID-19 cases can be affected by availability of medical resources, as too many cases at one point can overwhelm the health care system, leading to increasing risks of adverse outcome and death [18, 19]. This study was mainly focued on analyzing characteristics of COVID-19 cases and  identifying the risk factors for severe COVID-19 cases in order to prevent poor prognosis.

## Methods

### Setting, subjects, and data availability

The study setting was in 30 provinces outside Hubei Province in the mainland of China. Geographic classification into regions (eastern, central, and western) were based on socioeconomic level which were in accordance with the National Bureau of Statistics of China [20].

Subjects were symptomatic COVID-19 cases reported to the Chinese Center for Disease Control and Prevention (China CDC) COVID-19 online reporting system. The National Health Commission’s Protocol for Prevention and Control of COVID-19 issued by National Health Commission of the People’s Republic of China specified that county (district) level disease control and prevention agencies must complete an initial epidemiological investigation within 24 h upon receiving a report of a COVID-19 case and should submit investigation questionnaires to COVID-19 online reporting system. Epidemiological investigations include basic demographic data, initial symptoms at the time of diagnosis, laboratory findings and imaging features on admission, health-care-seeking behaviors and epidemiological history [21].

We included data of symptomatic cases of COVID-19 reported from January 19 to March 9, 2020. We matched information of cases in the COVID-19 online reporting system with their information in the National Notifiable Diseases Reporting System (NNDRS) based on national ID card number. Then we linked demographic data, date of symptom onset, date of diagnosis, and final clinical severity with epidemiological history, clinical manifestations, laboratory findings, radiographic features, and underlying diseases in the online reporting system. We then created a de-identified and unified dataset for all analyses.

The information in our study were from NNDRS and COVID-19 case reporting system. Analyses of these data are routine work, which are involved with relative divisions in China CDC.

### Definitions

Case definitions were based on Guidelines on Diagnosis and Treatment of Patients with COVID-19, which classifies cases as mild, moderate, severe, and critical [22]. Mild cases have mild symptoms without radiographic findings of pneumonia. Moderate cases have fever or cough with radiographic findings of pneumonia. Severe cases were those with shortness of breath, respiratory rate (RR) over 30/min, resting blood oxygen saturation less than 93%, and PaO_2_/FiO_2_ less than 300 mm Hg. Cases with radiographic findings of pneumonia progressing more than 50% in 24–28 h were considered severe cases. Critical cases had dyspnea with need for mechanical ventilation, shock, multiple organ failure, and required ICU care. The clinical severity classification in NNDRS is dynamic, which is revised by clinical doctors based on the progression of the cases, and being the most severe situation of cases finally. We categorized mild and moderate cases as non-severe cases, and categorized severe and critical cases as severe cases. Date of symptom onset was the onset date of fever, cough, or other symptoms of COVID-19, and was self-reported. Date of diagnosis was when the individual was diagnosed with COVID-19. All cases were laboratory confirmed by Real-Time reverse transcriptase Polymerase Chain Reaction (RT-PCR) assay. Early abnormal imaging included multiple small speckled shadows and interstitial changes with an obvious peripheral distribution, and bilateral multilobar ground-glass opacification and infiltration [22]. Laboratory tests and chest radiograph or Computed Tomography (CT) on admission were not mandatory in the case epidemiology investigation questionnaire, thus some data of laboratory findings and imaging features were unavailable.

Exposure history was determined by epidemiology history, travel or living in Wuhan or other areas with cases reported, contact history with symptomatic individuals from Wuhan and its surrounding areas or other communities reporting cases, contact history with a confirmed case or asymptomatic infected person, history of treatment, and presence of clustered cases in family, workplace, kindergartens, or schools.

### Statistical methods

This study used descriptive statistics to analyze median [interquartile range (IQR)], frequencies and proportions. We used univariate logistic regression analyses to determine association of variables with severity of COVID-19. Variables with *P*-values < 0.05 in the univariate logistic analyses were included in multivariable logistic regression. We used forward selection of variables in the logistic regression model and determined adjusted odds ratios (a*OR*s*)* and their 95% confidence intervals (*CI*s). A two-sided α of less than 0.05 (*P* < 0.05) was considered statistically significant. Statistical analyses were made with SPSS (Version 22.0).

## Results

### Sociodemographic and epidemiologic characteristics

The objects for this study were12 647 confirmed cases of COVID-19 reported in 30 provinces outside Hubei Province in mainland China between January 19, 2020 and March 9, 2020. The median age of all cases was 45 years (IQR: 33–56 years); the median age of severe cases was 57 years (IQR: 46–68 years) and the median age of non-severe cases was 43 years (IQR: 32–54 years). There were more cases among males (52.0%) than females (48.0%), and 23.6% cases had at least one underlying chronic diseases, 8.0% had an exposure history (Table 1).

**Table 1:** The characteristics of COVID-19 cases based on surveillanc data from January 19 to March 19 in 2020 in China

Variables	All cases, *n *(%)	Severe cases, *n* (%)
Sex		
Male	6581 (52.0)	965 (58.1)
Female	6066 (48.0)	697 (41.9)
Age group, years		
0–19	826 (6.5)	6 (0.4)
20–39	4132 (32.7)	253 (15.2)
40–59	5241 (41.4)	686 (41.3)
≥ 60	2448 (19.4)	717 (43.1)
Living area		
Urban	6923 (54.7)	959 (57.7)
Rural	5724 (45.3)	703 (42.3)
Regions		
East	4860 (38.4)	590 (35.5)
Central	5351 (42.3)	730 (44.0)
West	2436 (19.3)	342 (20.6)
From symptom onset to diagnosis, days		
0–2	3811 (30.1)	272 (16.4)
3–5	3610 (28.5)	447 (26.9)
6–8	2583 (20.4)	451 (27.1)
≥ 9	2643 (20.9)	492 (29.6)
Underlying disease		
No	9664 (76.4)	927 (55.8)
Yes	2983 (23.6)	735 (44.2)
Exposure history		
No	1009 (8.0)	215 (12.9)
Yes	11 638 (92.0)	1447 (87.1)

**Fig. 1:**
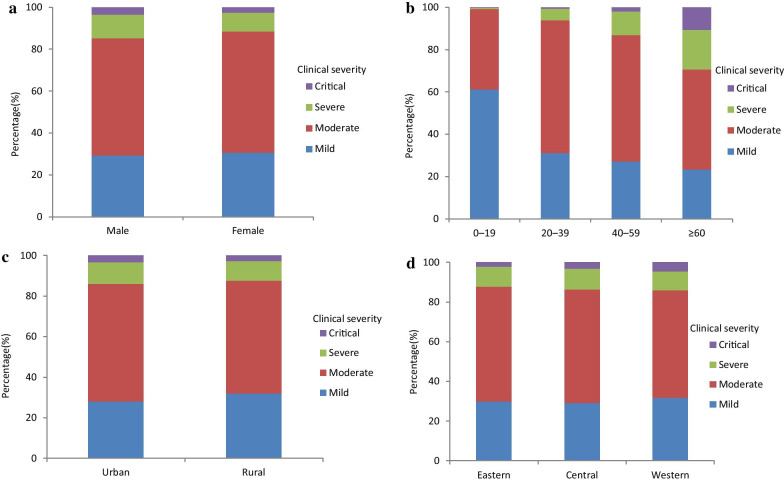
Clinical categories of different sociodemographic characteristics of COVID-19 cases. The data in this figure was surveillance data from January 19 to March 9 in 2020, from the NNDRS and COVID-19 online reporting system in China CDC

According to the definition of clinical classification of COVID-19, the 12 647 cases were categorized into 3788 (30.0%)mild cases, 7197 (56.9%) moderate cases, 1280 (10.1%)severe cases and 382 (3.0%)critical cases; 966 (58.1%) severe cases were male, 717 (43.1%) were ≥ 60 years, 6923 (54.7%) lived in urban area (Fig. 1).

### Characteristic of time from symptom onset to diagnosis

**Fig. 2:**
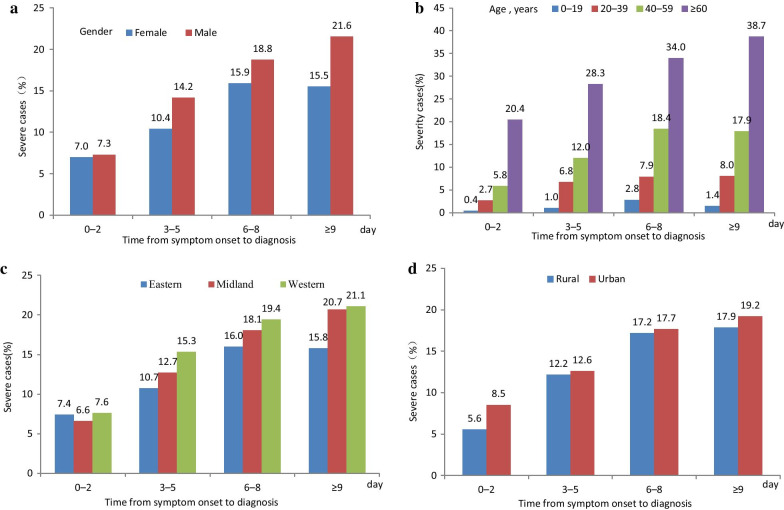
Severity of different characteristics of COVID-19 cases with different time from symptom onset to diagnosis. Data in this figure were surveillance data from January 19 to March 9 in 2020 from the NNDRS and COVID-19 online reporting system in China CDC

The median time from symptom onset to diagnosis was 4 days (IQR: 2–8 days) for the total cases, 6 days (IQR: 4–9) for severe cases, 5 days (IQR: 2–8) for non-severe cases. Regardless of gender, age group, region and residency, the proportion of severe cases increased with increasing time from symptom onset to diagnosis (Fig. 2).

### Proportion of severe COVID-19 cases progression

**Fig. 3:**
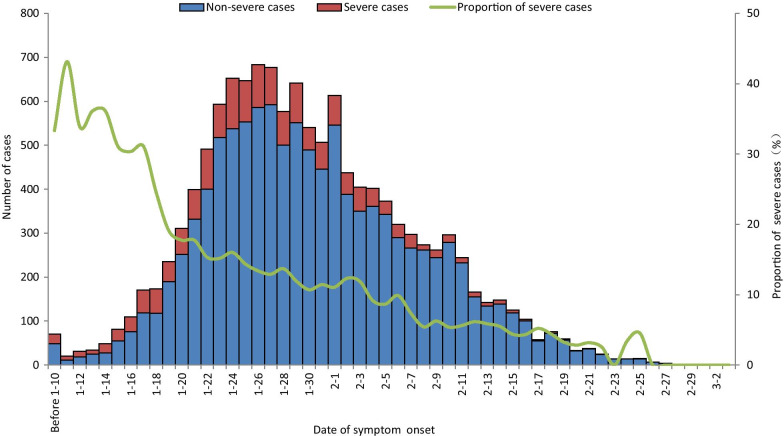
Distribution of severe and non-severe cases of COVID-19 with date of symptom onset. This figure shows the time course of the epidemic. The number and proportion of severe cases decreased after the epidemic peaked. Before January 23, 23.3% of cases were severe or critical, compared with 6.5% after February 5. Information in this figure was surveillance data from NNDRS and COVID-19 online reporting system

The proportion of severe cases decreased from 23.3% before January 23, 2020 to  6.5% after February 5, 2020 (Fig. 3).

### The risk factors of severe COVID-19 cases

The risk factors for developing severe COVID-19 were being male (a*OR* = 1.3, 95% *CI:* 1.2–1.5); fever (a*OR* = 2.3, 95% *CI:* 2.0–2.7), fatigue (a*OR* = 1.4, 95% *CI:* 1.2–1.6), cough (a*OR* = 1.3, 95% *CI:* 1.2–1.5), diabetes (a*OR* = 1.5, 95% *CI:* 1.2–1.8), hypertension (a*OR* = 1.5, 95% *CI:* 1.2–1.8) and chronic kidney disease (a*OR* = 2.5, 95% *CI:* 1.4–4.6); abnormal CT imaging (a*OR* = 1.6, 95% *CI:* 1.2–2.1), lymphocyte count decrease (a*OR* = 1.3, 95% *CI:* 1.1–1.5). With the increase of age, the risk for severity was gradually higher, < 20 years (a*OR* = 1), 20–39 years (a*OR* = 3.9, 95% *CI:* 1.8–8.4), 40–59 years (a*OR* = 7.6, 95% *CI:* 3.6–16.3), ≥ 60 years (a*OR* = 20.4, 95% *CI:* 9.5–43.7). With the increase of time from symptom onset to diagnosis, the risk of severity was gradually higher, 0–2 days (a*OR* = 1), 3–5 days (a*OR* = 1.4, 95% *CI:* 1.2–1.7), 6–8 days (a*OR* = 1.8, 95% *CI:* 1.5–2.1), ≥ 9 days (a*OR* = 1.9, 95% *CI:* 1.6–2.3) (Table 2).

**Table 2:** The risk factors for developing severe COVID–19 cases based on surveillance data from January 19 to March 9 in 2020 in China

Variable	Severe cases (*n*)/all cases (*N*) (%)	Crude *OR* (95% *CI*)	*P* value	a*OR* (95% *CI*)
Gender				
Female	697/6066 (11.5)	1		1
Male	965/6581 (14.7)	1.33 (1.2–1.5)	0.000	1.34 (1.2–1.5)
Age group, years				
0–19	6/826 (0.8)	1		1
20–39	253/4132 (6.1)	7.63 (3.6–16.2)	0.000	3.90 (1.8–8.4)
40–59	686/5241 (13.1)	17.62 (8.3–37.2)	0.000	7.63 (3.6–16.3)
≥ 60	717/2448 (29.3)	48.46 (22.9–102.5)	0.000	20.42 (9.5–43.7)
Living area				
Rural	703/5724 (12.3)	1		1
Urban	959/6923 (13.9)	1.15 (1.0–1.3)	0.010	0.94 (0.8–1.1)
Region				
East	590/4860 (12.1)	1		1
Central	730/5351 (13.7)	1.14 (1.0–1.3)	0.022	1.12 (1.0–1.3)
West	342/2436 (14.0)	1.18 (1.0–1.4)	0.022	1.40 (1.2–1.6)
The symptom onset to diagnosis, days				
0–2	272/3811 (7.1)	1		1
3–5	447/3610 (12.4)	1.84 (1.6–2.2)	0.000	1.41 (1.2–1.7)
6–8	451/2583 (17.5)	2.75 (2.4–3.2)	0.000	1.79(1.5–2.1)
≥ 9	492/2643 (18.6)	2.98 (2.5–3.5)	0.000	1.91(1.6–2.3)
Fever				
No	265/4376 (6.1)	1		1
Yes	1397/8271 (16.9)	3.14 (2.7–3.6)	0.000	2.30(2.0–2.7)
Fatigue, joint or muscle soreness				
No	1034/9397 (11.0)	1		1
Yes	628/3250 (19.3)	1.94 (1.7–2.2)	0.000	1.33(1.2–1.5)
Cough or respiratory symptoms				
No	520/5597 (9.3)	1		1
Yes	1142/7050 (16.2)	1.75 (1.6–1.9)	0.000	1.40(1.2–1.6)
Gastrointestinal symptoms				
No	1495/11712 (12.8)	1		1
Yes	167/935 (17.9)	1.48 (1.3–1.8)	0.000	1.09(0.9–1.3)
Headache				
No	1446/11308 (12.8)	1		1
Yes	216/1339 (16.1)	1.31 (1.1–1.5)	0.001	1.15(1.0–1.4)
Conjunctival congestion				
No	1658/12625 (13.1)	1		
Yes	4/22 (18.2)	1.47 (0.5–4.4)	0.487	
White blood cell count (10^9^/L)^a^				
4–10	1088/7981 (13.6)	1.0		1
< 4	356/2477 (14.4)	1.06 (0.9–1.2)	0.351	1.03(0.9–1.2)
> 10	87/400 (21.8)	1.76 (1.4–2.3)	0.000	1.47(1.1–2.0)
Unavailable	131/1789 (7.4)	0.51 (0.4–0.6)	0.000	0.54(0.2–1.5)
Lymphocyte count (10^9^/L)^b^				
0.8–3.5	1029/8632 (11.9)	1		1
< 0.8	431/1752 (24.6)	2.41 (2.1–2.7)	0.000	1.28 (1.1–1.5)
> 3.5	62/444 (14.0)	1.20 (0.9–1.6)	0.197	1.22 (0.9–1.7)
Unavailable	140/1819 (7.8)	0.62 (0.5–0.8)	0.000	1.72 (0.8–3.6)
Lymphocyte percentage(%)^c^				
20–40	690/6084 (11.3)	1		1
< 20	739/3441 (21.5)	2.14 (1.9–2.4)	0.000	1.19 (1.0–1.4)
> 40	100/1333 (7.5)	0.63 (0.5–0.8)	0.000	0.77 (0.60–1.0)
Unavailable	133/1789 (7.5)	0.63 (0.5–0.8)	0.000	1.22 (0.4–3.8)
Neutrophil percentage (%)^d^				
40–75	914/7928 (11.5)	1		1
< 40	117/944 (12.4)	1.09 (0.9–1.3)	0.433	1.27 (1.0–1.6)
> 75	498/1984 (25.1)	2.57 (2.3–2.9)	0.000	1.55 (1.3–1.9)
Unavailable	133/1791 (7.5)	0.62 (0.5–0.8)	0.000	0.98 (0.3–2.8)
Abnormal X-ray				
No	326/2576 (12.7)	1		1
Yes	239/1319 (18.1)	1.53 (1.3–1.8)	0.000	1.29 (1.1–1.6)
Unavailable	1097/8752 (12.5)	0.99 (0.9–1.1)	0.883	0.96 (0.8–1.1)
Abnormal CT imaging				
No	326/2576 (12.7)	1		1
Yes	239/1319 (18.1)	3.21 (2.4–4.3)	0.000	1.57(1.2–2.1)
Unavailable	1097/8752 (12.5)	2.14 (1.6–2.9)	0.000	1.53(1.1–2.2)
Underlying diseases				
No	927/9664 (9.6)	1		1
Yes	735/2983 (24.6)	3.08 (2.8–3.4)	0.000	0.88 (0.7–1.1)
Diabetes				
No	1447/12 098 (12.0)	1		1
Yes	215/549 (39.2)	4.73(4.0–5. 7)	0.000	1.96 (1.6–2.4)
Hypertension and other cardiovascular and cerebrovascular diseases				
No	1195/11126 (10.7)	1		1
Yes	467/1521 (30.7)	3.68 (3.3–4.2)	0.000	1.46 (1.2–1.8)
Lung diseases				
No	1574/12350 (12.8)	1		1
Yes	88/297 (29.6)	2.88 (2.2–3.7)	0.000	1.42 (1.1–1.9)
Chronic liver diseases				
No	1648/12 550 (13.1)	1		
Yes	14/97 (14.4)	1.12 (0.6–2.0)	0.707	
Chronic kidney diseases				
No	1637/12 594 (13.0)	1		1
Yes	25/53 (47.2)	5.97 (3.5–10.3)	0.000	2.50 (1.4–4.6)
Exposure history				
No	215/1009 (21.3)	1		1
Yes	1447/11 638 (12.4)	0.53 (0.5–0.6)	0.000	0.63 (0.5–0.8)

^a^ The normal range of the white blood cell count is 4 × 10^9^/L to 10 × 10^9^/L

^b^ The lymphocyte count normal range is 0.8 × 10^9^/L to 3.5 × 10^9^/L

^c^ The lymphocyte percentage normal range is 20% to 40%

^d^ The normal range of percent neutrophils is 40% to 75% [23]

## Discussion

COVID-19 are bringing huge burden on public health globally, and decreasing the severity of cases would reduce the medical resources and case fatality.  This study found risk factors included being male, older age, symptoms including fever, cough and fatigue at the time of diagnosis, lymphopenia, abnormal CT imaging at the time of diagnosis, hypertension, diabetes, and chronic disease, which were consistent with some reports [4, 12, 15, 19, 24]. Meanwhile, our study found that having exposure history and timely diagnosis decreased the risks for developing severe cases. These findings are helpful for predicting and preventing the poor prognosis of COVID-19 cases, moreover providing evidence for further making strategy for controlling COVID-19.

Regarding the gender in our study, males had higher risk of developing severe COVID-19 than females, which is consistent with some reports [4, 24]. The differences between different gender may be related to underlying biological differences, diversity of immune responses, sex hormones and a combination of behavioral or lifestyle risk factors [25–27]. Furthermore, several comorbidities, which disproportionally occur in men, likely contribute to worse COVID-19 outcomes [28]. The clinical situation of COVID-19 cases varied by age. We found that the elderly tended to have severe illness, while younger individuals had less  severe cases. This might due to the lower immunity and higher prevalence rate of comorbidities in elderly people [29]. In contrast with adults, young generation less than 20 years that mainly include school students and children were less frequently exposed to the main sources of virus transmission, as their movement was limited by interventions such as closure of schools [11, 30]. And some reported that most infected children were less likely to be symptomatic or develop severe symptoms, as well as the percentage of patients who were treated in the ICU or received invasive mechanical ventilation was increased for the elderly [31, 32].

Similar to SARS-CoV and MERS-CoV infections, the most common symptoms of COVID-19 were fever and cough, but the absence of fever in Covid-19 was more frequent than SARS-CoV and MERS-CoV infection [34, 35, 36]. Fever is a reaction  activated by the immune systems to resist pathogens, and cough might indicate the defense against invaders [4]. Consitent with some reports, hyperpyrexia more often appeared  in older patients than that in young and middle age patients with COVID-19 [37]. Also the hyperpyrexia associated with adverse outcomes in patients with COVID-19, which might be caused by SARS-CoV-2 related brain injury, exuberant immune response, and thrombus formation reported by relative study [38]. 

Laboratory examinations can provide essential assistance to predict severity of COVID-19 [39]. The most common laboratory test abnormality was lymphopenia. [28, 40] which associated with final COVID-19 severity [1, 10]. Chest imaging played a key role in the early evaluation by allowing rapid triage of dyspneic patients [41]. Early in the progression of this disease, CT imaging findings in approximately 15% of individuals and chest radiograph findings in approximately 40% of individuals can be normal [42]. Similar to our result, previous study demonstrated that initial chest CT abnormalities would indicate a poor prognosis [41].

As for COVID-19, the average time from exposure to symptom onset is 5 days, and 97.5% of people who develop symptoms do so within 11.5 days [42, 43]. Early diagnosis and prompt medical care help relieve symptoms and signs, prevent illness aggravation and lessen severity [32, 33]. A study indicated that presymptomatic transmission is a major contributor to the spread of SARS-CoV-2, early identification and diagnosis of cases could prevent more infections [44], moreover, can prevent the poor prognosis.

Our study showed that underlying diseases were associated with increasing risks of severity of COVID-19, and that 55.8% of severe cases had at least one chronic disease, a lower percentage than that the 68% found in a study of critical patients infected with SARS-CoV-2 admitted to ICU [45]. SARS-CoV-2 infection can affect the cardiovascular system and can cause acute kidney failure [46, 47]. Meanwhile, chronic underlying conditions can cause metabolic disorders and weaken immune systems, all of these factors place patients with hypertension and chronic kidney failure at greater risk of severe SARs-CoV-2 infection [48, 49]. The Guidelines on Diagnosis and Treatment of Patients with COVID-19 also indicates that people with these underlying diseases are at high risk of severity [1].

A history of travel to epidemic area was important for early case finding and management. According to the Protocol for Prevention and Control of COVID-19, persons with travel history to Wuhan or other high risk communities were paid more attention to be screened as a high-risk population and strictly followed up, medical workers should to increase awareness of people with exposure histories [50, 51]. In the early phase of the epidemic (before January 23), 23.3% were severe, and most cases reported from other provinces outside Hubei were from Hubei [52]. To control the epidemic, provinces outside Hubei fortified quarantine and management of people from Hubei, and ensuring home-medical-observation for 14 days and admission to designated hospitals with related symptoms arise [53, 54]. These control and prevention measures were instrumental in shortening time from symptom onset to diagnosis, allowing people with exposure histories to receive early detection and early medical care, which could be explanations for our finding that having exposure histories reduced the risks of  developing severe COVID-19 cases.

There are several limitations in our study. One is that data in our study were from January to March in 2020, the epidemiological and clinical characteristics might be different as for the mutation of the virus. Besides, the symptoms at the time of diagnosis and date of symptom onset were based on self-report, there might be recall bias. Third, the laboratory and imaging testing were not mandatory in admission, so some data were unavailable. Finally, we did not have hospitalised treatment records which might also influence the progression of illness.

## Conclusions

Our study with large sample size explored the risk factors for developing severe cases of COVID-19. Our study found early diagnosis and having exposure history reduced the risks of COVID-19 severity. Meanwhile, the risk factors of developing severe COVID-19 including male, older age, fever, fatigue, cough, hypertension, diabetes, and chronic kidney disease, which are very helpful to predict and prevent developing severe cases of COVID-19. Therefore, we should pay more attention to cases with these risk factors, and avert poor prognosis.

## Data Availability

According to the requirement of the NNDRS in China CDC, original data can be used only by our researchers and cannot be provided to others.

## Abbreviations

COVID-19: Coronavirus Disease 2019
SARS-CoV-2: Severe Acute Respiratory Syndrome Coronavirus 2
MERS-CoV: Middle East Respiratory Syndrome Coronavirus
SARS-CoV: Severe Acute Respiratory Syndrome Coronavirus
MERS: Middle East Respiratory Syndrome
SARS: Severe Acute Respiratory Syndrome
ICU: Intensive Care Unit
WHO: World Health Organization
China CDC: Chinese Center for Disease Control and prevention
NNDRS: National Notifiable Diseases Reporting System
RR: Respiratory Rate
CT: Computed Tomography
RT-PCR: Real-time reverse transcriptase Polymerase Chain Reaction
a*OR*: adjusted Odds Ratio
*CI*: Confidence Interval
