# Correction to: Risk factors for developing severe COVID-19 in China: an analysis of disease surveillance data

**DOI:** 10.1186/s40249-021-00868-7

**Published:** 2021-06-02

**Authors:** Meng-Jie Geng, Li-Ping Wang, Xiang Ren, Jian-Xing Yu, Zhao-Rui Chang, Can-Jun Zheng, Zhi-Jie An, Yu Li, Xiao-Kun Yang, Hong-Ting Zhao, Zhong-Jie Li, Guang-Xue He, Zi-Jian Feng

**Affiliations:** 1 Division of Infectious Diseases, Chinese Center for Disease Control and Prevention, Beijing, China; 2 National Institute for Communicable Disease Control and Prevention, Chinese Center for Disease Control and Prevention, Beijing, China; 3 National Immunization Program, Chinese Center for Disease Control and Prevention, Beijing, China; 4 National Institute for Viral Disease Control and Prevention, Chinese Center for Disease Control and Prevention, Beijing, China

## Correction to: Infect Dis Poverty (2021) 10:48 10.1186/s40249-021-00820-9

Following publication of the original article [1], two errors were found in the article:

In the Conclusion section of the Abstract, the sentence "early case identification and prompt medical care" need to be deleted, the whole sentence should be:

Our study showed the risk factors for developing severe COVID-19 with large sample size, which included being male, older age, fever, cough, fatigue, delayed diagnosis, hypertension, diabetes, chronic kidney disease. Based on these factors, the severity of COVID-19 cases can be predicted. So cases with these risk factors should be paid more attention to prevent severity.

In the Conclusion section of the article, the word ‘disease’ was missed, the whole sentence should be:

Our study with large sample size explored the risk factors for developing severe cases of COVID-19. Our study found early diagnosis and having exposure history reduced the risks of COVID-19 severity. Meanwhile, the risk factors of developing severe COVID-19 including male, older age, fever, fatigue, cough, hypertension, diabetes, and chronic kidney disease, which are very helpful to predict and prevent developing severe cases of COVID-19. Therefore, we should pay more attention to cases with these risk factors, and avert poor prognosis.

The original paper has been updated.
